# Development and Performance Evaluation of a High-Temperature-Resistant Salt-Responsive Micro-Crosslinked Polymer Gel Filtration Loss Reducer

**DOI:** 10.3390/gels12070564

**Published:** 2026-06-25

**Authors:** Fengfeng Xiao, Yuhao Xia, Wushuo Liu, Jingping Liu, Yuanwei Sun

**Affiliations:** 1 Drilling Fluid Technology Service Company, CNPC Chuanqing Drilling Engineering Co., Ltd., Chengdu 610051, China; xiaoff_sc@cnpc.com.cn (F.X.); xiayuhao_sc@cnpc.com.cn (Y.X.); 2 School of Petroleum Engineering, China University of Petroleum—East China (UPC), Qingdao 266580, China; z25020076@s.upc.edu.cn (W.L.); liujingping20@126.com (J.L.)

**Keywords:** water-based drilling fluid, filtration loss reducer, micro-crosslinking, salt-responsive, high-temperature resistance

## Abstract

To address the difficulty in controlling the filtration performance of water-based drilling fluids under high-temperature and high-salinity conditions during the drilling of deep and ultra-deep wells, a salt-responsive micro-crosslinked polymer gel filtration loss reducer, designated LZX, was developed. The synthesis employed 2-acrylamido-2-methylpropane sulfonic acid (AMPS), N,N-dimethylacrylamide (DMAA), dimethyldiallylammonium chloride (DMDAAC), and a betaine monomer containing an unsaturated double bond as monomers, with polyethylene glycol diacrylate (PEGDA) introduced as a crosslinker. Experimental results showed that the product structure matched the design expectations, and the thermal decomposition temperature of the main molecular chain exceeded 290 °C, indicating good thermal stability. At 220 °C under saturated salt conditions, a dosage of 2.5 wt% LZX maintained the API filtration loss at 5.8 mL and the HPHT filtration loss at 28.6 mL. Comparative experiments at different temperatures demonstrated that LZX exhibited superior filtration control performance compared to the commercial high-temperature filtration reducer Driscal Temp and Driscal D. The micro-crosslinked structure of LZX enhanced the rigidity of the molecular chains, raising the upper limit of its thermal resistance. Rheological and viscosity-average molecular weight measurements revealed that LZX exhibited typical antipolyelectrolyte behavior in high-salinity environments—the molecular chains tended to extend and the filtration reduction capability was accordingly maintained—preliminarily achieving a functional transition from passive salt tolerance to active salt responsiveness. LZX is expected to support the construction of high-performance water-based drilling fluids with high temperature and high salt resistance for future deep-earth drilling.

## 1. Introduction

As global oil and gas exploration and development continue to advance into deep and ultra-deep formations, the encountered extreme conditions such as high temperature, high pressure, and high-salinity evaporite beds are becoming increasingly severe [1,2]. When the well depth exceeds 5000 m, the temperature can reach above 200 °C, while the probability of encountering high-pressure brine zones and complex salt-gypsum beds increases significantly [3,4]. Under such conditions, the regulation of the filtration performance of water-based drilling fluids faces severe challenges. High temperatures aggravate the thermal degradation and hydrolysis of the molecular chains of polymer filtration loss reducers, and also lead to a thinning of the hydration layer of clay particles and a decrease in their dispersion stability. High concentrations of salt ions compress the electrical double layer, weakening the electrostatic adsorption between the polymer and clay [5,6]. In salt solutions, the molecular chains of conventional polymer filtration loss reducers are prone to a coiled conformational transition, causing a sharp loss of their viscositying and filtration reduction capabilities, and the filtration loss often increases severalfold [7]. Although existing acrylamide-based polymer filtration loss reducers have undergone continuous modification, they still commonly suffer from structural instability, adsorption failure, and attenuation of filtration reduction ability under the combined action of high temperature and high salt [8]. These issues make it difficult to meet the stringent requirements of long-term stability for drilling fluids in deep drilling. Therefore, how to break through the design limitations of traditional temperature- and salt-resistant polymers and develop a novel polymer filtration loss reducer with stable structure and performance under high-temperature and high-salinity conditions has become a critical technical bottleneck that urgently needs to be resolved.

To address the problem of polymer filtration loss reducer failure under high-temperature and high-salinity environments, current research mainly focuses on three dimensions: optimizing the molecular structure of linear polymers, regulating the aggregated state structure of polymers, and utilizing nanocomposite technology [6]. In terms of molecular structure optimization, the introduction of bulky groups such as rigid aromatic rings or heterocyclic monomers [9] into the side chains can enhance the rigidity of the molecular chains, restrict the thermal motion of chain segments [10], and thereby improve the thermal stability of the polymer. With respect to the regulation of the aggregated state structure, the formation of a micro-crosslinked network through moderate covalent crosslinking [11] can inhibit the violent thermal motion and thermal degradation of molecular chains at high temperatures, allowing such filtration loss reducer to remain effective above 200 °C. Meanwhile, the introduction of strongly hydratable functional groups such as sulfonic acid groups and N-vinylpyrrolidone [12,13], or nanostructural units such as cage-like oligomeric silsesquioxane [14], exploits their hydration capacity and steric hindrance effects to delay the compression of molecular chains by salt ions. As a result, additives with strong temperature resistance and a certain filtration reduction capability even in saturated brine or high-calcium environments have been developed. Ren et al. [10] developed a micro-crosslinked amphoteric polymer gel, PAAT, which maintained the API filtration loss within 8 mL under 210 °C; Rong et al. [11] reported a micro-crosslinking copolymer with a filtration loss below 10 mL at 220 °C; Li et al. [14] synthesized an organic–inorganic copolymer, PAAD, which exhibited a filtration loss of 15.4 mL under high-calcium conditions at 180 °C; and Ma et al. [15] developed a betaine-type polymer filtration loss reducer, which maintained the API filtration loss within 10 mL at 200 °C. However, the performance of most of these products still deteriorates under the combined stress of higher temperatures and saturated brine, and it appears that their salt-responsive mechanisms rely largely on passive salt tolerance rather than active chain extension in high-salinity environments.

The above strategies face the inherent challenge of balancing thermal stability, salt adaptability, and water solubility. Rigid groups improve thermal stability but often impair polymer dissolution and dispersion. Micro-crosslinking can prevent excessive chain coiling, yet high crosslinking density compromises solubility and dispersion [16]. Highly hydratable groups enhance salt resistance, but under high salinity, molecular chains tend to collapse and lose hydration, causing performance to deteriorate with increasing salinity [17]. Most existing studies focus on enhancing the passive tolerance of polymers to harsh environments, failing to fundamentally address the key scientific issue of molecular chain coiling and reduction of the hydrodynamic radius in salt solutions. Therefore, there is an urgent need to develop a novel design concept: leveraging the salt-responsive characteristics of the polymer to enable it to actively extend, to some extent, in high-salinity environments, thereby maintaining or even enhancing its filtration reduction performance.

To address the adaptability to high-temperature and high-salinity environments, zwitterionic polymers reported in existing studies are typically constructed by copolymerizing cationic and anionic monomers [18]. However, under high-temperature and high-salinity conditions, the random distribution of oppositely charged units along the chain makes their electrostatic pairing easily decoupled by the intensified ionic atmosphere at elevated temperatures, leading to a significant weakening of the antipolyelectrolyte effect, and the molecular chain struggles to effectively extend under high salinity [14]. To this end, this study proposes a design strategy based on a micro-crosslinked structure and salt-responsive behavior. A betaine structural unit is introduced into the molecular chain, containing equimolar quaternary ammonium cations and sulfonic or carboxylic acid anions within the same pendant group, forming a stable internal salt form. The distance between the positive and negative charge centers is fixed and unaffected by external ion exchange [15,19]. This structure is expected to enable the polymer to exhibit antipolyelectrolyte behavior in high-temperature, high-salinity aqueous environments, thereby providing a novel approach to transition from passive salt tolerance to actively utilizing the salt environment to regulate molecular chain extension. Additionally, a moderate micro-crosslinked network formed by PEGDA inhibits thermal motion and degradation at high temperatures, enhancing thermal stability [20], while preserving water solubility and dispersibility.

Based on the above design concept, a novel filtration loss reducer, designated LZX, which possesses both a micro-crosslinked network and salt-responsive betaine units, was prepared via free-radical copolymerization using AMPS, DMAA, DMDAAC, and a betaine monomer containing an unsaturated double bond as raw materials, with polyethylene glycol diacrylate as a crosslinker. The chemical structure of LZX was confirmed by means of Fourier-transform infrared spectroscopy and elemental analysis, and its thermal stability and molecular weight characteristics were characterized by means of thermogravimetric analysis and gel permeation chromatography. The effects of LZX dosage on the rheology and filtration loss of the base slurry were systematically investigated, and its filtration reduction performance under high-temperature and various salt contamination conditions was evaluated. The variations in viscosity-average molecular weight and solution viscosity of the polymer under different salt concentrations were analyzed using an Ubbelohde viscometer and rheological measurements. Combined with Zeta potential, particle size distribution, scanning electron microscopy, and adsorption experiments, the mechanism of LZX in high-temperature and high-salinity environments was explored. This study is expected to provide a novel molecular design reference for the development of high-performance drilling fluid additives for deep, complex formations.

## 2. Results and Discussion

### 2.1. Characterization

#### 2.1.1. Fourier Transform Infrared Spectroscopy Analysis

**Figure 1:**
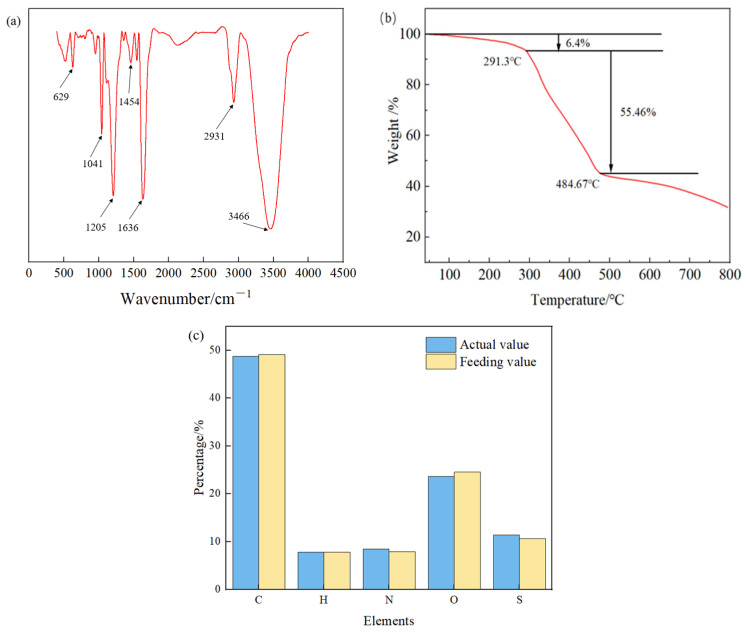
Characterization of LZX: (**a**) FTIR spectrum, (**b**) Thermogravimetric curve, (**c**) elemental analysis of LZX.

The infrared spectrogram of LZX is shown in Figure 1a. The characteristic peaks at 629 cm^−1^ correspond to the O–H bending vibration and the C–S bond deformation vibration. The symmetric stretching vibration absorption band of the sulfonic acid group (–SO_3_^−^) appears at 1041 cm^−1^ [21]. The peaks at 1205 cm^−1^ and 1454 cm^−1^ are assigned to the C–O bending vibration and the C–N stretching vibration, respectively [22,23]. The characteristic peak of the C=O group is observed at 1636 cm^−1^ [24]. The –CH_2_– asymmetric stretching vibration peak appears at 2931 cm^−1^, and the secondary amine N–H stretching vibration is observed at 3466 cm^−1^ [25]. The FTIR results indicate that all the monomers used in the synthesis participated in the reaction, confirming that the obtained filtration loss reducer is identical to the target product.

#### 2.1.2. Thermogravimetric Analysis

Figure 1b shows the thermogravimetric analysis (TGA) curve of LZX. The weight loss process of LZX can be roughly divided into three stages. The first stage, from 40 °C to 291.3 °C, is the physical dehydration stage, with a mass loss of approximately 6.4%. This is mainly due to the thermal evaporation of adsorbed and bound water, which desorbs from the polymer. The second stage, from 291.3 °C to 484.67 °C, is the main chain decomposition stage, with a mass loss of approximately 55.46%. As the temperature continues to rise, the functional groups in the molecular chains begin to break upon heating, the main and side chains undergo scission, and part of the main chain also starts to decompose. Above 484.67 °C, the carbonization stage begins, and the remaining LZX gradually decomposes into carbonaceous residue. Comprehensive analysis shows that the thermal decomposition of LZX starts at 291.3 °C, indicating relatively good thermal stability.

#### 2.1.3. Elemental Analysis

Elemental analysis results are presented in Figure 1c. The measured contents of C, H, N, O, and S in LZX are 48.72%, 7.83%, 8.41%, 23.65%, and 11.39%, respectively, which are in good agreement with the theoretical values calculated from the feed ratios (C 49.13%, H 7.78%, N 7.88%, O 24.54%, S 10.66%), with deviations for all elements within 1.0%. Based on the measured elemental proportions, the actual molar ratio of the four monomers (AMPS:DMAA:DMDAAC:betaine monomer) in the product was back-calculated to be approximately 9.8:5.0:3.9:2.9, which closely corresponds to the feed molar ratio of 10:5:4:3. Taken together, the FTIR and elemental analysis results confirm that the synthesized product is the target copolymer LZX.

#### 2.1.4. Gel Permeation Chromatography Analysis

The molecular weight results of LZX are presented in Table 1. The experimental results show that the polydispersity index (D) of LZX is 1.7907, indicating a relatively moderate molecular weight distribution, which suggests reasonable control over the polymerization process of this filtration loss reducer. Its weight-average molecular weight (Mw) is 385,000, falling within the medium molecular weight range. The moderate molecular weight facilitates the dissolution and dispersion of the polymer in water, providing favorable conditions for salt-responsive chain extension in high-salinity environments, while avoiding the insufficient dissolution issues commonly encountered with high-molecular-weight polymers. Furthermore, the moderate chain length allows effective adsorption onto the surface of clay particles to form a stable adsorption layer, while preventing excessive intermolecular entanglement caused by overly long chains, thereby maintaining good rheological properties.

### 2.2. Evaluation of Rheological and Filtration Properties

#### 2.2.1. Effect of Filtration Loss Reducer on Rheological and Filtration Properties of Base Slurry

**Figure 2:**
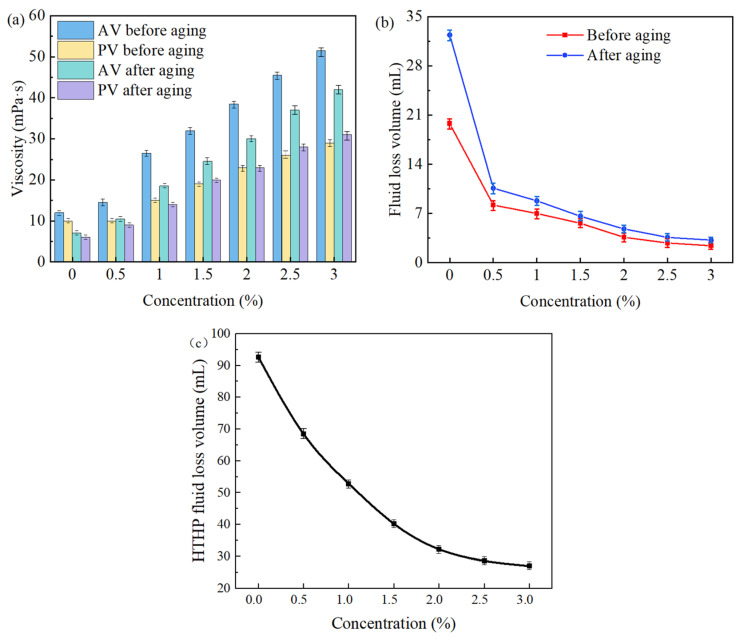
Effect of Filtration Loss Reducer on Rheological and Filtration Properties of Drilling Fluid Prior to and After Aging: (**a**) rheological properties; (**b**) FL_API_; (**c**) FL_HTHP_.

The changes in rheological and filtration properties of the drilling fluid base slurry as a function of LZX concentration, before and after aging at 220 °C for 16 h, are presented in Figure 2.

With increasing LZX dosage, both the apparent viscosity and plastic viscosity of the base slurry show an increasing trend. After aging at 220 °C for 16 h, the viscosity of the base slurry at each dosage decreases, but the viscosity values still maintain a positive correlation with LZX dosage. Figure 2b shows that the addition of LZX significantly reduces the API filtration loss of the base slurry. Without LZX, the API filtration loss of the base slurry is 19.8 mL. When the LZX dosage reaches 2.5 wt%, the filtration loss decreases to 2.8 mL, corresponding to a filtration reduction efficiency of 85.9%, demonstrating a pronounced filtration reduction effect. After aging at 220 °C for 16 h, the filtration loss of the base slurry alone increases to 32.4 mL, while that of the base slurry with 2.5 wt% LZX is only 3.6 mL, yielding a filtration reduction efficiency of 88.9%. When the LZX dosage is further increased to 3.0 wt%, the reduction in filtration loss becomes marginal. To facilitate a uniform dosage for subsequent performance comparisons, 2.5 wt% was selected as the optimal dosage of LZX for further studies. Figure 2c shows that the HPHT filtration loss of the base slurry is significantly reduced upon addition of LZX. At a dosage of 2.5 wt%, the HPHT filtration loss of the base slurry decreases from 92.6 mL to 28.6 mL, corresponding to a filtration reduction efficiency of 69.1%.

The above results indicate that with increasing dosage of the filtration loss reducer, both the API and HPHT filtration losses of the base slurry gradually decrease, and the excellent filtration reduction capability is maintained even after aging.

#### 2.2.2. Evaluation of High-Temperature Resistance of Filtration Loss Reducer

**Figure 3:**
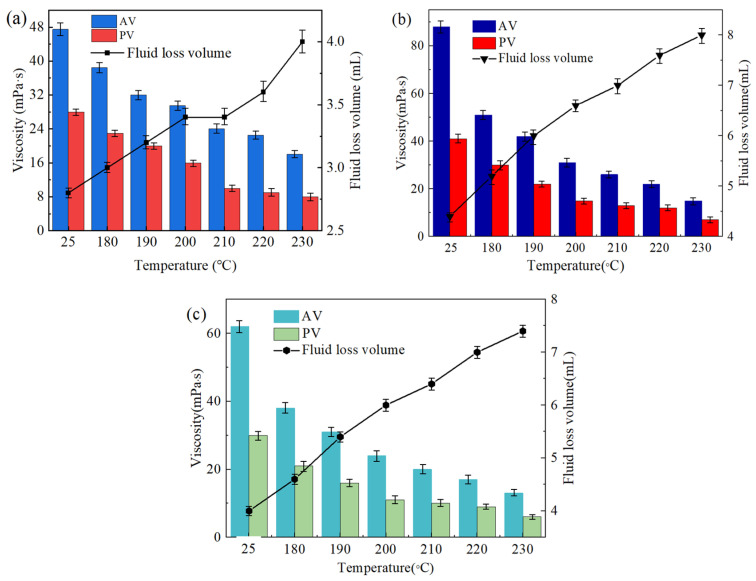
Thermal stability evaluation of different filtration loss reducers after aging at different temperatures for 16 h: (**a**) LZX; (**b**) Driscal Temp, (**c**) Driscal D.

The rheological and filtration performance of the base slurry containing 2.5 wt% LZX, Driscal Temp, and Driscal D [26], after aging at various temperatures for 16 h, is presented in Figure 3.

The experimental results show that with increasing aging temperature, the viscosity of the LZX-containing drilling fluid decreases slightly and the filtration loss increases gradually, though the overall changes remain relatively small. After aging at 220 °C, the API filtration loss is 3.6 mL; upon further aging at 230 °C, the API filtration loss is only 4 mL. Compared with the two commercial Driscal series products, the drilling fluids containing Driscal Temp and Driscal D both exhibit higher viscosity than the LZX-containing system, but their filtration losses are also markedly larger. Among them, Driscal Temp displays the most pronounced viscosifying effect but poor filtration control, with an API filtration loss of 7.6 mL after aging at 220 °C. Driscal D shows slightly lower viscosity than Driscal Temp and a modest reduction in filtration loss, with an API filtration loss of 7.2 mL at 220 °C, still far exceeding that of LZX.

The above results demonstrate that both Driscal series products function primarily as viscosifiers with limited high-temperature filtration reduction capability. In contrast, LZX exerts a minor impact on the rheological properties of the drilling fluid and maintains effective filtration control within the temperature range of 220–230 °C, exhibiting excellent temperature resistance.

#### 2.2.3. Evaluation of Salt Resistance of Filtration Loss Reducer

**Figure 4:**
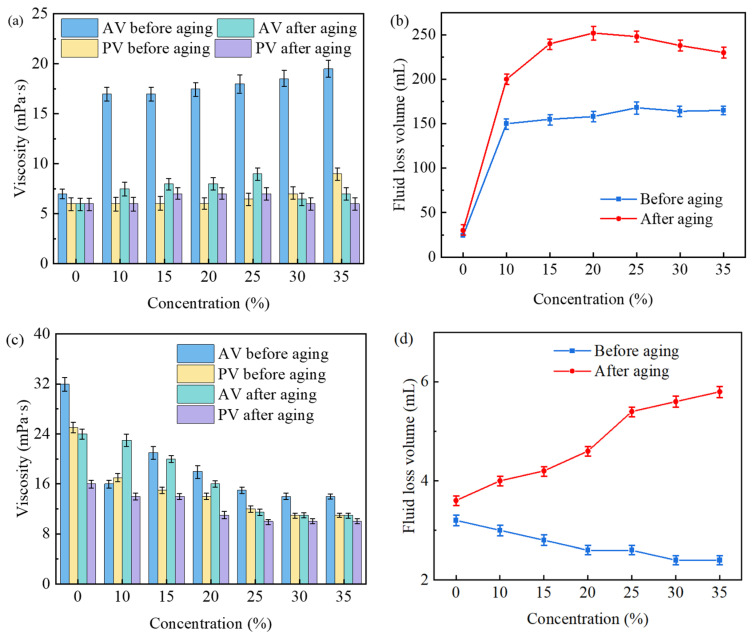
Evaluation of salt resistance of the base slurry and LZX-containing drilling fluid before and after aging: (**a**) base slurry rheological properties; (**b**) base slurry filtration performance; (**c**) LZX rheological properties; (**d**) LZX filtration performance.

The rheological and filtration performance of the base slurry and the drilling fluid with 2.5 wt% LZX, before and after aging at various NaCl concentrations, is presented in Figure 4.

Compared with the viscosity of the base slurry (Figure 4a), after the addition of LZX (Figure 4c), the viscosity shows a slight decreasing trend with increasing NaCl dosage, and the magnitude of change is relatively small, further indicating that LZX has a mild impact on the rheological properties of the drilling fluid. In terms of filtration performance, the filtration loss of the base slurry (Figure 4b) increases continuously with increasing NaCl dosage, exceeding 150 mL when the NaCl dosage reaches 10 wt%. However, after adding 2.5 wt% LZX (Figure 4d), the filtration loss before aging decreases with increasing NaCl dosage, and remains below 6 mL across all NaCl dosages. This trend is consistent with the aforementioned antipolyelectrolyte viscosity-enhancing behavior of the LZX solution, providing experimental support for the salt-responsive characteristic of LZX: in high-salinity environments, the molecular chains tend to extend and the filtration reduction capability is effectively maintained. After high-temperature aging, the filtration loss increases somewhat with increasing NaCl dosage. This is primarily attributed to two factors: first, the high-salinity environment compresses the electrical double layer of clay particles, weakening the repulsive force between particles and promoting their agglomeration, thereby reducing the effective adsorption of LZX molecular chains onto the clay surface; second, high temperature causes a certain degree of thermal damage to the molecular chain structure of LZX, leading to a partial reduction in its filtration reduction efficiency. Nevertheless, the filtration loss after aging remains below 6 mL across all NaCl dosages.

In summary, LZX has a minor effect on the rheology of the drilling fluid while significantly reducing filtration loss, demonstrating good salt resistance.

### 2.3. Mechanism Study

#### 2.3.1. Zeta Potential Analysis

**Figure 5:**
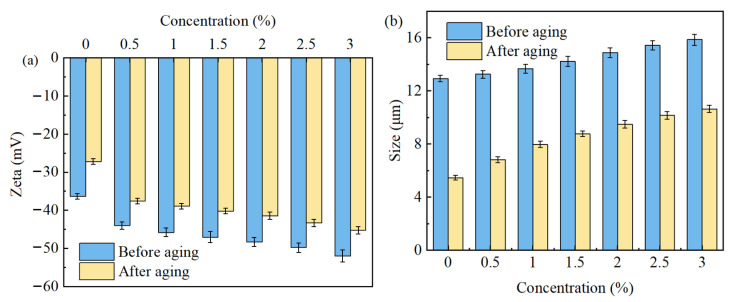
Characterization of LZX: (**a**) Zeta potential variation and (**b**) particle size distribution.

As shown in Figure 5a, with increasing LZX dosage, the absolute value of the Zeta potential of the drilling fluid before and after aging exhibits an increasing trend. When the LZX dosage reaches 2 wt%, the change in Zeta potential tends to level off. This is because the LZX polymer molecular chains adsorb onto the surface of bentonite particles, thickening the hydration layer of the particles and enhancing the electrostatic repulsion between them, thereby increasing the absolute value of the Zeta potential [27]. Consequently, LZX maintains the dispersion stability of clay particles in the system and inhibits agglomeration by improving the degree of clay particle hydration, thereby achieving filtration loss reduction.

#### 2.3.2. Particle Size Analysis

As can be seen from Figure 5b, with increasing LZX dosage, the particle size of the base slurry before and after aging generally shows an increasing trend. The main reason is that after LZX adsorbs onto the clay particle surface, it increases the thickness of the hydration layer, leading to an increase in the measured apparent particle size of the particles.

#### 2.3.3. Mechanism of Salt Response

**Figure 6:**
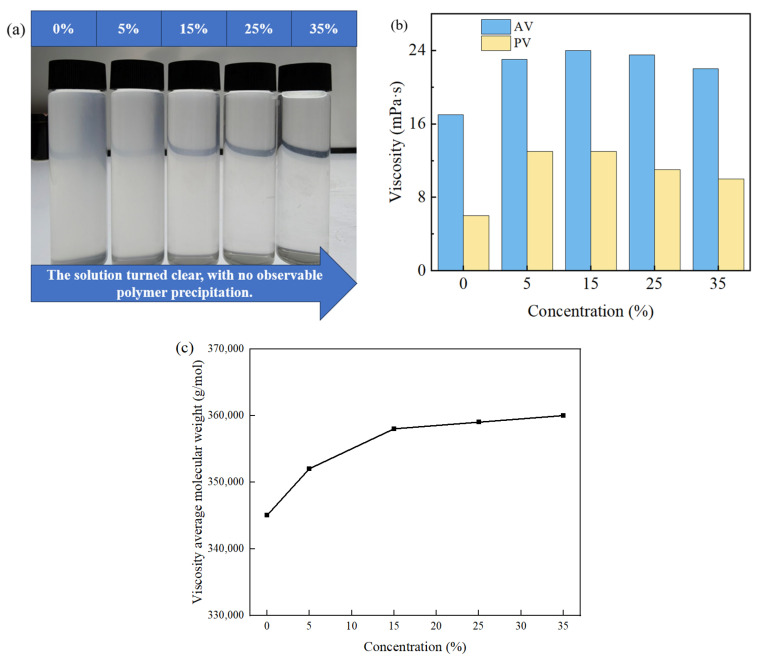
(**a**) Transparency variation of LZX aqueous solution under different NaCl dosages; (**b**) Rheological property variation of LZX aqueous solution under different NaCl dosages; (**c**) Viscosity-average molecular weight under different NaCl dosages.

The photographs visually demonstrate the transparency evolution of a 2.5 wt% LZX aqueous solution under different NaCl dosages. As shown in Figure 6a, in the absence of NaCl and at 5% NaCl, the LZX solution appears slightly turbid. This is attributed to intramolecular electrostatic association between the positively and negatively charged groups within the betaine structure in the absence of external free electrolyte shielding, causing the polymer segments to adopt a compactly coiled conformation that forms colloidal domains capable of scattering incident light.

As the NaCl dosage increases from 5% to 15%, the solution transparency significantly improves. This transition is a typical macroscopic manifestation of the salt-responsive characteristic of LZX: the salt ions screen the electrostatic attraction between the positive and negative charges on the betaine side groups, disrupting the intramolecular association and inducing a conformational transition of the polymer molecular chains from a collapsed state to an extended conformation. When the NaCl dosage is further increased to 35%, the solution remains transparent, with no recurrence of turbidity or polymer precipitation. This indicates that under high-salinity conditions, the LZX molecular chains can maintain their extension continuously, without the chain contraction or salting-out precipitation commonly observed for conventional zwitterionic polymers at high salinities. This stable salt-responsive behavior directly confirms that LZX possesses the ability to actively extend and hydrate in high-salinity environments, providing intuitive evidence for its excellent filtration reduction performance in saturated brine drilling fluids.

As shown in Figure 6b, the viscosity variation clearly demonstrates the unique antipolyelectrolyte viscosity-enhancing effect of the LZX aqueous solution. In deionized water, the positive and negative charges on the betaine side groups drive intramolecular electrostatic association, causing chain segments to collapse, resulting in a small hydrodynamic volume and a low initial viscosity. As the NaCl dosage increases from 0 to 15%, the salt ions gradually screen the internal salt bonds, disrupting the intramolecular association. The chain conformation transitions from a collapsed to an extended state, the hydrodynamic volume increases, and the solution viscosity begins to rise. With further increase in NaCl dosage, the solution viscosity does not drop sharply due to charge screening, as is commonly observed for conventional zwitterionic polymers, but instead remains at a relatively high level and levels off. This indicates that the molecular chains of LZX can maintain a stable extended conformation even at extremely high salt concentrations, without undergoing dehydration-induced collapse. This excellent performance is closely related to the micro-crosslinked network within the molecular chains: moderate crosslinking enhances chain rigidity, inhibits excessive chain collapse under high-salinity conditions, and thus significantly broadens the salinity range over which the antipolyelectrolyte effect operates.

To verify the above conformational transition at the molecular level, the viscosity-average molecular weight of LZX was measured using an Ubbelohde viscometer under varying NaCl concentrations (Figure 6c). It should be noted that the viscosity-average molecular weight determined by means of Ubbelohde viscometry reflects the intrinsic viscosity of the polymer and can be used to characterize the extension state of the molecular chains in solution. When the molecular chains are well extended with a larger hydrodynamic volume, the intrinsic viscosity increases, resulting in a correspondingly higher measured viscosity-average molecular weight. The variation in this value reflects the trend of conformational transition of the chains, rather than an actual change in the chemical molecular weight of the polymer [28]. As shown in Figure 6c, in pure water, the viscosity-average molecular weight of LZX was approximately 345,000, lower than the weight-average molecular weight determined by means of GPC (385,000), consistent with the coiled state of the molecular chains in low-salinity environments. As the NaCl concentration increased from 0% to 5%, the viscosity-average molecular weight rose slightly to 352,000; at 15%, it reached 358,000; thereafter, it stabilized at 359,000 and 360,000 at 25% and 35%, respectively, essentially leveling off. This “slight increase followed by stabilization” trend is consistent with the theoretical expectations of the antipolyelectrolyte effect: after salt ions screen the internal salt association, the molecular chains transition from a coiled to an extended conformation, increasing the hydrodynamic volume and thus the intrinsic viscosity; once chain extension reaches its limit, the viscosity-average molecular weight no longer changes appreciably with further increases in salt concentration. These results provide experimental support for the mechanism whereby salt ions drive the conformational transition of the molecular chains.

The experimental evidence from the above three aspects—the transparency transition, the increase in viscosity-average molecular weight, and the rise in rheological parameters—corroborates one another and collectively indicates that LZX possesses stable and sustained chain extension capability in high-salinity environments. This provides multi-scale experimental support, from the molecular to the macroscopic level, for its ability to maintain effective filtration reduction and favorable dispersion stability in saturated brine drilling fluids.

#### 2.3.4. Adsorption Amount Analysis

**Figure 7:**
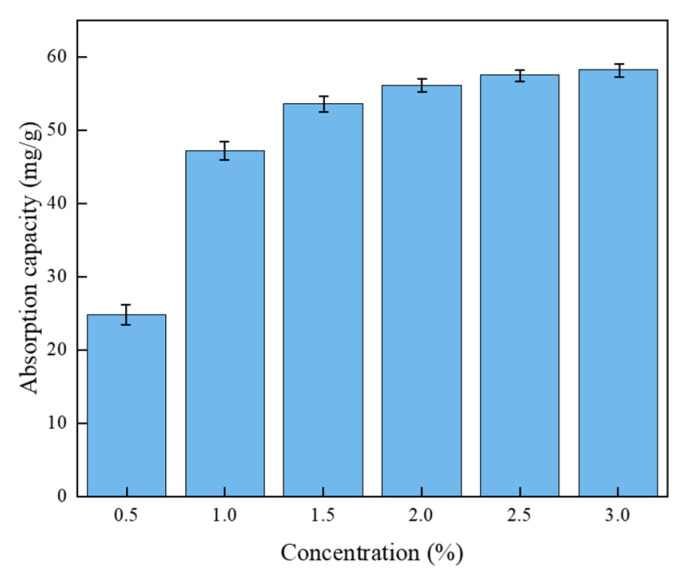
Analysis of LZX adsorption amount.

The influence of varying LZX concentration on the adsorption amount of LZX onto clay surfaces in the drilling fluid is presented in Figure 7.

As shown in Figure 7, with increasing LZX dosage, the adsorption amount of LZX onto the surface of clay particles gradually increases. When the LZX dosage reaches 1.5 wt%, the adsorption amount curve tends to level off, indicating that the adsorption of LZX molecular chains onto the clay surface gradually approaches saturation. Further increasing the dosage yields only a limited increase in adsorption amount, and the molecular chains are already in an efficient interaction region. The TOC method employed in this study is intended to compare the adsorption trend at different dosages and thereby provide a preliminary assessment of the adsorption behavior of LZX on clay surfaces [28]. Taken together with the aforementioned Zeta potential and particle size distribution results, LZX effectively adsorbs onto the surface of clay particles, thereby increasing the absolute value of the Zeta potential, thickening the hydration layer, inhibiting particle aggregation, and promoting the formation of a dense filter cake, ultimately reducing the filtration loss of the drilling fluid effectively.

#### 2.3.5. Mud Cake Micromorphology Analysis

**Figure 8:**
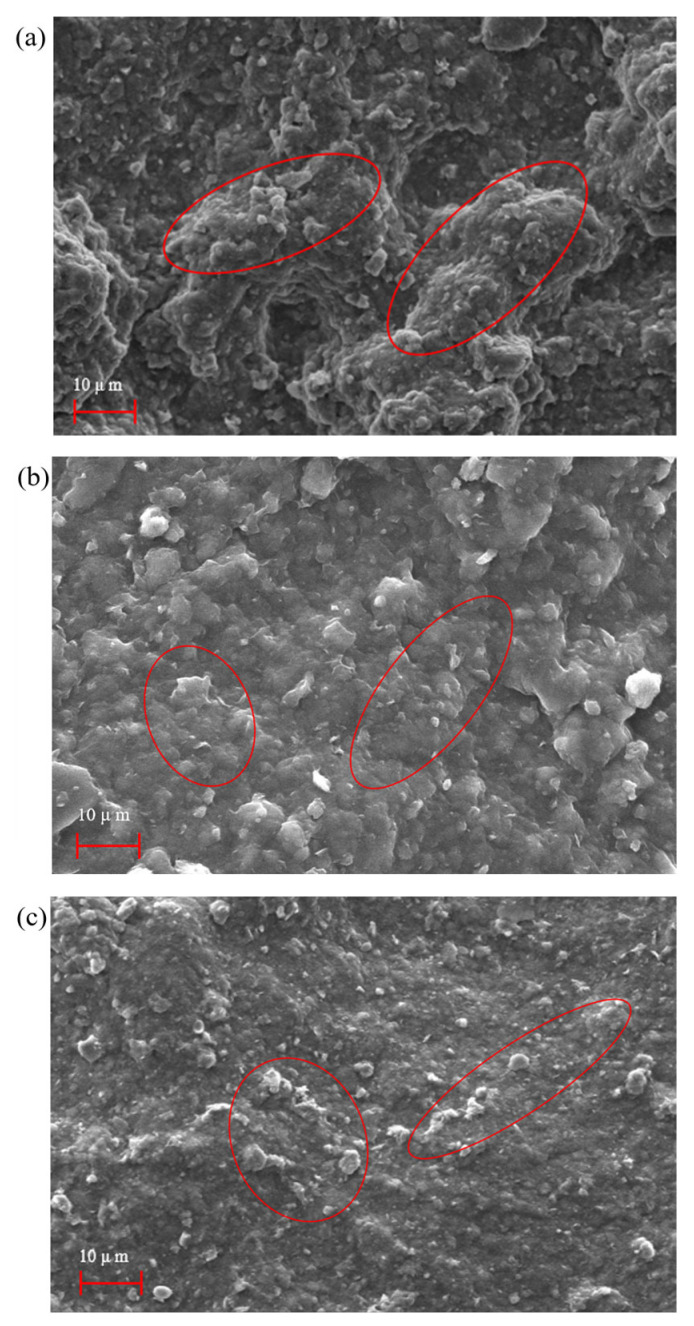
Mud cake morphology analysis. (**a**) Base slurry, (**b**) base slurry + 2.5 wt% LZX, (**c**) base slurry + 2.5 wt% LZX + 36 wt% NaCl.

Figure 8 presents scanning electron microscopy (SEM) images of filter cakes from different systems after aging at 220 °C: Figure 8a shows the filter cake of the base slurry; Figure 8b shows the filter cake of the drilling fluid containing 2.5 wt% LZX; and Figure 8c shows the filter cake of the saturated salt drilling fluid containing 2.5 wt% LZX.

As can be seen from Figure 8a, the surface of the base slurry filter cake is uneven, with significant aggregation of clay particles and an increase in particle size. Numerous micropores are distributed among the large particles, forming a connected pore network, which is the direct cause of the high filtration loss of the base slurry. After the addition of LZX (Figure 8b), the filter cake surface becomes smooth and flat, the clay particle size is significantly reduced, the particles are more densely packed, and the micropores are markedly diminished. This indicates that LZX can effectively adsorb onto the surface of clay particles, thickening the hydration layer and thereby inhibiting the severe aggregation of clay particles at high temperatures. Under saturated salt conditions (Figure 8c), some clay particles and salt-crystal agglomerates are visible in the filter cake. Nevertheless, the filtration loss reducer still effectively adsorbs onto the clay particle surface, and no large pores or cracks are observed on the filter cake surface. The cake is overall smooth and dense, with significantly improved quality. The above results further demonstrate the excellent filtration reduction performance of LZX under high-temperature and high-salinity conditions.

#### 2.3.6. Mechanism Analysis of the Filtration Loss Reducer

**Figure 9:**
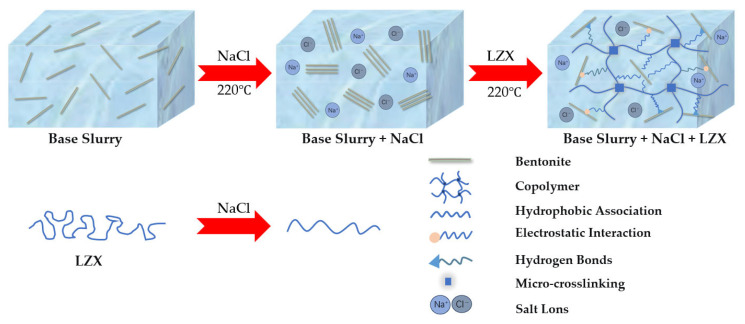
Schematic diagram of filtration loss reducer.

As shown in Figure 9, in the base slurry system, clay particles are in a relatively dispersed state. Upon addition of NaCl, the electrical double layer of the clay particles is compressed in the high-salinity environment, significantly reducing the electrostatic repulsion between particles and causing severe clay particle aggregation. After the addition of LZX, the betaine structure within the LZX molecule endows it with a unique salt-responsive characteristic: as the salt concentration increases, the salt ions screen the intramolecular electrostatic association between the positive and negative charges, inducing a conformational transition of the polymer molecular chains from a collapsed state to an extended state. The extended molecular chains expose more adsorption sites, enabling firm adsorption onto the clay particle surfaces through hydrogen bonding and electrostatic interactions. Meanwhile, the micro-crosslinked network introduced by PEGDA enhances the rigidity of the molecular chains, inhibits excessive chain coiling at high temperatures, and imparts a certain degree of elasticity.

## 3. Conclusions

1. A salt-responsive filtration loss reducer with a micro-crosslinked structure, designated LZX, was successfully prepared via free-radical polymerization using AMPS, DMAA, DMDAAC, and a betaine monomer as raw materials, with PEGDA introduced as a crosslinker. FTIR and elemental analysis results demonstrated that the product is the target copolymer. GPC measurements gave a weight-average molecular weight of 385,000 and a polydispersity index of 1.79, indicating a relatively moderate molecular weight distribution. TGA showed that the thermal decomposition temperature of the main chain exceeds 291.3 °C, demonstrating good thermal stability.
2. LZX exhibits a mild viscosifying effect in drilling fluids and outstanding high-temperature filtration reduction performance. At a dosage of 2.5 wt%, after aging at 220 °C for 16 h, the API filtration loss is only 3.6 mL, and the HPHT filtration loss is significantly reduced from 92.6 mL (base slurry) to 28.6 mL. Compared with two commercially available filtration loss reducers, Driscal Temp and Driscal D, LZX shows superior filtration reduction capability and a lower impact on viscosity at high temperatures, with an effective temperature resistance up to 220 °C.
3. LZX exhibits typical antipolyelectrolyte salt-responsive behavior. Ubbelohde viscometer and rheological measurements demonstrated that as the salt concentration increases, the viscosity-average molecular weight rises slightly from 345,000 to 360,000 before leveling off, and the molecular chains transition from a collapsed to an extended conformation, thereby enhancing the adsorption stabilization and bridging effect on clay particles. Under saturated salt conditions, the API filtration loss can be controlled at 2.4 mL. Although the filtration loss increases slightly after high-temperature aging, it remains at 5.8 mL, preliminarily achieving the design advantage of transitioning from passive salt tolerance to active salt responsiveness.
4. Mechanistic studies indicate that LZX effectively controls filtration loss by increasing the absolute value of the Zeta potential on clay particle surfaces, thickening the hydration layer, inhibiting face-to-face aggregation at high temperatures, and forming a smooth and dense filter cake. The synergistic design of the micro-crosslinked structure and the betaine internal salt achieve a favorable balance among high-temperature stability, salt adaptability, and water solubility, providing a new technical pathway for the development of high-temperature and high-salt resistant water-based drilling fluid additives for deep drilling. The excellent comprehensive performance under conditions of 220 °C and saturated salt is expected to support the safe and efficient implementation of future ultra-deep well drilling projects.

## 4. Materials and Methods

### 4.1. Materials and Instruments

N,N-Dimethylacrylamide (DMAA, AR), 2-acrylamido-2-methyl-1-propanesulfonic acid (AMPS, AR), and polyethylene glycol diacrylate (PEGDA, AR) were purchased from Shanghai Haohong Biomedical Technology Co., Ltd. (Shanghai, China). Dimethyldiallylammonium chloride (DMDAAC, AR) and the betaine monomer containing an unsaturated double bond were obtained from Saien Chemical Technology (Shanghai, China) Co., Ltd. Driscal D, Driscal Temp was provided by Chevron Phillips Chemical Co., Ltd. (The Woodlands, TX, USA). Sodium hydroxide (AR), ammonium persulfate (APS, AR), sodium carbonate (AR), and sodium chloride (AR) were supplied by Sinopharm Chemical Reagent Co., Ltd. (Shanghai, China). Drilling fluid bentonite was purchased from Shandong Huawei Bentonite Co., Ltd. (Weifang, China).

The main instruments used were as follows: Fourier transform infrared spectrometer (IRTRacer-100, Shimadzu, Kyoto, Japan), thermogravimetric analyzer (TGA2, Mettler Toledo, Greifensee, Switzerland), gel permeation chromatograph (1260 GPC, Agilent, Santa Clara, CA, USA), six-speed rotational viscometer (ZNN-D6, Qingdao Tongchun, Qingdao, China), Elementar Unicube elemental analyzer (Elementar, Langenselbold, Germany), API filter press (SD6A, Qingdao Tongchun, Qingdao, China), roller oven (GW300-PLC, Qingdao Tongchun, Qingdao, China), laser nanoparticle size analyzer (Zetasizer Nano ZS90, Malvern, Worcestershire, UK), laser particle size analyzer (Mastersizer 3000, Malvern, Worcestershire, UK), high-pressure high-temperature filter press (GGS71-B, Qingdao Xusheng Petroleum Instrument Co., Ltd., Qingdao, China), and total organic carbon analyzer (TOC-L, Shimadzu, Kyoto, Japan).

### 4.2. Molecular Structure Design of the Filtration Loss Reducer

**Figure 10:**
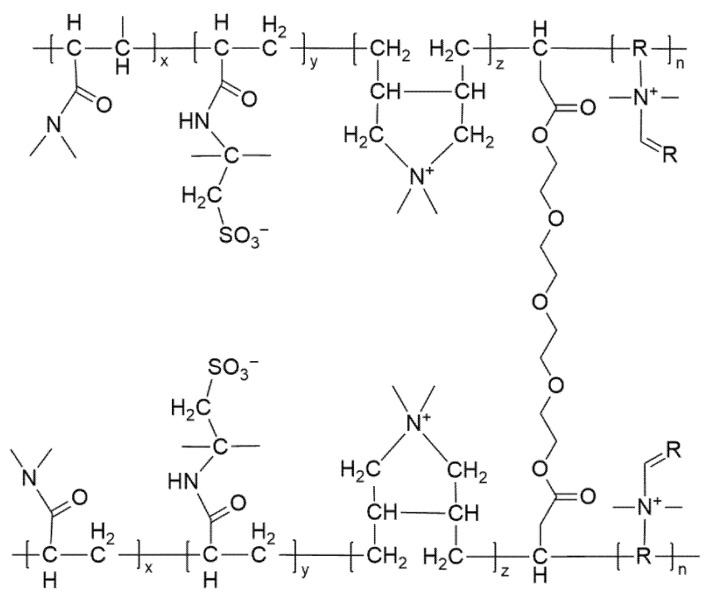
Molecular structure design of the filtration loss reducer.

The molecular structure design of the filtration loss reducer is shown in Figure 10. Based on literature research and analysis of the failure mechanisms of drilling fluid additives [29,30,31], a design strategy for the filtration loss reducer was established. DMAA, as the main chain monomer, provides thermal stability and hydrogen-bonding adsorption capacity to clay. The sulfonic acid group in AMPS endows the polymer with excellent temperature and salt resistance, maintaining the chain in an extended state under high-temperature and high-salinity conditions. The betaine monomer, with its fixed positive and negative charge centers unaffected by external ion exchange, exhibits an antipolyelectrolyte effect in high-salinity environments, prompting the molecular chain to extend and exposing more adsorption sites through segment unfolding, thereby enhancing multi-point binding to the clay surface. DMDAAC provides a high density of positive charges, strengthening electrostatic binding to the clay surface, increasing the thickness of the hydration layer, and inhibiting clay dispersion at high temperatures. PEGDA [32,33,34], as a crosslinker, forms a moderately crosslinked network, endowing the filtration loss reducer with rigidity and elasticity, allowing it to fill micro-nano pores of the filter cake and reduce permeability [31,35]. Through synergistic effects, the drilling fluid filtration loss is effectively reduced under ultra-high temperature and saturated brine conditions, forming a dense filter cake and ensuring wellbore stability.

### 4.3. Synthesis of the Filtration Loss Reducer

The monomer mole ratio was AMPS:DMAA:DMDAAC:betaine monomer = 10:5:4:3, with a total monomer concentration of 40 wt%. An aqueous solution polymerization method was adopted. AMPS was added to deionized water and stirred until completely dissolved. The pH was adjusted to 7–8 using a 30 wt% NaOH solution. Then, DMAA, DMDAAC, and the betaine monomer were sequentially added to the solution and stirred to dissolve. After stirring for ten minutes, the resulting solution was transferred to a round-bottom three-neck flask equipped with a stirring device. Nitrogen gas was introduced, and the water bath temperature was set to 70 °C with a stirring speed of 350 rpm. When the temperature of the liquid in the flask reached the reaction temperature, PEGDA (0.5 wt% relative to the total monomer mass) and an ammonium persulfate solution (0.5 wt%) were added. The reaction was allowed to proceed for 6 h. After completion, the product was washed several times with a mixed solution of anhydrous ethanol and acetone, then dried in a constant-temperature oven and crushed [36,37]. The resulting product was designated as the filtration loss reducer LZX.

### 4.4. Characterization of the Filtration Loss Reducer

1. Fourier Transform Infrared Spectroscopy (FTIR): LZX was mixed with KBr and pressed into translucent pellets. The Fourier transform infrared absorption spectrum of LZX was measured in the range of 4000 to 400 cm^−1^ using a Fourier transform infrared spectrometer.
2. Thermogravimetric Analysis (TGA): Approximately 8 mg of LZX was placed in a crucible. The crucible with the sample was placed in a thermogravimetric analyzer, and the thermogravimetric curve of LZX was measured from 40 °C to 600 °C under a nitrogen atmosphere at a heating rate of 5 °C/min.
3. The elemental composition of LZX was analyzed using the Elementar Unicube elemental analyzer (Elementar, Langenselbold, Germany).
4. Gel Permeation Chromatography (GPC) Analysis: The molecular weight of LZX was determined using a gel permeation chromatograph. First, 20 mg of LZX was added to 10 mL of water (deionized water containing 0.1 M NaNO_3_) and allowed to stand at 25 °C for 24 h, with occasional stirring during this period to promote dissolution of LZX. Then, the solution was filtered through a microfiltration membrane (0.22 μm) to remove insoluble polymer [38]. Finally, the clarified solution was injected into the gel chromatography column for measurement.

### 4.5. Evaluation of Rheological and Filtration Properties

Deionized water (400 mL) was added to a mixing cup. Anhydrous sodium carbonate (0.69 g) and bentonite (20.0 g) were sequentially added with continuous stirring. The mixture was stirred for a total of 20 min and then sealed and aged for 24 h at room temperature to obtain the base slurry for evaluation. For performance evaluation, the rheological properties were measured using a six-speed rotational viscometer [39], and the filtration loss was measured using an API filter press. The HPHT filtration loss was measured at 180 °C using a high-pressure high-temperature filter press [40]. Hot-rolling aging was conducted at various temperatures for 16 h using a roller oven.

1. Different amounts of LZX were added to the base slurry and stirred for 20 min. The rheological and filtration properties of the drilling fluid were measured before aging and after aging at 220 °C for 16 h. The HPHT filtration loss of the base slurry was measured at 180 °C using a high-pressure high-temperature filter press.
2. Evaluation of Temperature Resistance: LZX, Driscal Temp and Driscal D was added to the base slurry at a dosage of 2.5 wt%, stirred for 20 min, and then aged at different temperatures for 16 h. The rheological and filtration properties of the base slurry after aging at different temperatures were measured.
3. Evaluation of Salt Resistance: LZX (2.5 wt%) and various amounts of NaCl were added to the base slurry. The rheological and filtration properties of the slurry were measured before and after aging at 220 °C.

### 4.6. Mechanism Study

1. Zeta Potential Analysis: Different amounts of LZX were added to the base slurry. The Zeta potential of the base slurry with varying LZX dosages was measured before aging and after aging at 220 °C using a laser nanoparticle size analyzer.
2. Particle Size Analysis: Different amounts of LZX were added to the base slurry. The particle size of the base slurry with varying LZX dosages was measured before and after aging using a laser particle size analyzer.
3. Salt-Responsive Characteristics: LZX (2.5 wt%) was added to deionized water, followed by the addition of various amounts of NaCl. Changes in solution transparency and viscosity were observed and measured to evaluate the salt-responsive performance of the filtration loss reducer. Separately, LZX was dissolved in deionized water with varying NaCl concentrations (0%, 5%, 15%, 25%, and 35%) to prepare dilute solutions. The efflux time was measured using an Ubbelohde [41] capillary viscometer at a constant temperature of 25 °C. The intrinsic viscosity was determined by the dilution extrapolation method, and the viscosity-average molecular weight at each salt concentration was calculated using the Mark–Houwink equation.
4. Adsorption Amount Analysis: The adsorption amount of LZX onto the clay surface under different LZX dosages was measured using a total organic carbon (TOC) analyzer. LZX (2.5 wt%) was dissolved in deionized water to prepare solutions of varying concentrations, which were then added to 4 wt% bentonite base slurry and stirred for 24 h to reach adsorption equilibrium. A 50 mL aliquot of each drilling fluid sample was centrifuged at 3000 rpm for 15 min. The lower clay sediment was collected, washed twice with deionized water to remove loosely attached polymer on the surface, dried under vacuum at 60 °C, and then ground to 200 mesh. Untreated bentonite (30 mg), pure LZX, and bentonite treated with different LZX dosages were each heated to 900 °C for TOC measurement. The adsorption amount was calculated using Equation (1) [42]:

(1) $$M_{\varphi }=\frac{M_{3}-M_{1}}{M_{2}}\;\times 1000$$

where $M_{\varphi }\;$ (mg/g) is the adsorption amount of LZX, $M_{1}\;$ (g) is the TOC value of 30 mg of untreated bentonite, $M_{2}\;$ (g) is the TOC value of 30 mg of LZX, and $\;M_{3}\;$ (g) is the TOC value of 30 mg of LZX-treated bentonite.

5. Microscopic Morphology Analysis of Filter Cake: API filter cakes were collected from the base slurry, the drilling fluid containing 2.5 wt% LZX, and the saturated salt drilling fluid containing 2.5 wt% LZX after aging at 220 °C for 16 h. The filter cakes were dried, and the surface microstructure was observed using a scanning electron microscope (SEM).

## Figures and Tables

**Table 1:** Molecular Weight Data of LZX.

Mw	Mn	Mz	Mp	Mz + 1	Mw/Mn
385,000	215,000	615,000	295,000	850,000	1.7907

## Data Availability

The data presented in this study are available on request from the corresponding author.

## References

[1] Wang J., Sun J.-S., Huang X.-B., Lv K.-H., Xie S.-X. (2026). High-Temperature Resistant Zwitterionic Filtration Loss Reducer for Brine-Based Drill-In Fluids. Energy Fuels.

[2] Abdulhadi D., Ali J.A., Hama S.M. (2025). Advanced Techniques for Improving the Production of Natural Resources from Unconventional Reservoirs: A State-of-the-Art Review. Energy Fuels.

[3] Guo X.S., Hu D.F., Li Y.P., Duan J.B., Zhang X.F., Fan X.J., Duan H., Li W.C. (2019). Theoretical Progress and Key Technologies of Onshore Ultra-Deep Oil/Gas Exploration. Engineering.

[4] Wang Y.L., Jiang B.Y., Lan J.C., Xu N., Sun J.S., Meng L.T. (2020). Synthesis and properties of a high-performance environment-friendly micro-nano filtration reducer. Rsc. Adv..

[5] Sun J., Yang J., Lu K., Bai Y., Liu J., Huang X. (2025). Research status and prospects of drilling fluid technology for tight oil and gas. Acta Pet. Sin..

[6] Deng X., Lyu K., Li J., Sun J., Fan J., Liao T., Huang N. (2025). Research status and development trend of polymer fluid loss reducer for ultra-deep drilling fluid. Chem. Ind. Eng. Prog..

[7] Liu L.M., Sun J.S., Wang R., Qu Y.Z., Liu F., Yang J., Cheng R.C., Gao S.F., Huang H.J. (2022). Synthesis of a new high temperature and salt resistant zwitterionic filtrate reducer and its application in water-based drilling fluid. Colloids Surf. A Physicochem. Eng. Asp..

[8] Lin L., Li Z., Gu H., Xiong G.X., Luo Y.H., Pu D., Luo P.Y. (2024). Acrylamide-Based Polymer/Laponite Composite as a Filtration Reducer in Water-Based Drilling Fluids. Energy Fuels.

[9] Li Z., Liang D., Peng B., Yang S., Yang H. (2016). Synthesis and Application Filtrate Loss Reducer HRF with Temperature Resistance and Salt Tolerance. Oilfield Chem..

[10] Chen D., Luo H., Tie C., Li W., Zhao X. (2013). Summary on Fluid Loss Additive Used for Drilling Fluids. Oilfield Chem..

[11] Rong K., Yang Y., Xu S., Deng P., Pu X., Luo X. (2018). Preparation and Performance Evaluation of Micro-crosslinking Polymer Filtration Reducer with High Temperature Resistance. Oilfield Chem..

[12] Zhang J., Li H., Yang S., Chen H., Zhang Z., Abeuzar M. (2020). Research and application of new high temperature-reducing filter loss agent. Appl. Chem. Ind..

[13] Jain R., Mahto V. (2015). Evaluation of polyacrylamide/clay composite as a potential drilling fluid additive in inhibitive water based drilling fluid system. J. Pet. Sci. Eng..

[14] Li X., Duan M., Deng Z., Xian L., Su Y., Xu Z. (2023). Preparation and Performance Evaluation of Composite Fluid Loss Reducer Containing Caged Nanoparticles. Oilfield Chem..

[15] Ma X.P., Yang M., Zhang M. (2020). Synthesis and properties of a betaine type copolymer filtrate reducer. Chem. Eng. Process.-Process Intensif..

[16] Yang J., Dong T.F., Yi J.T., Jiang G.C. (2024). Development of Multiple Crosslinked Polymers and Its Application in Synthetic-Based Drilling Fluids. Gels.

[17] Moukoko A.D.K., Yang L.L., Jiang G.C., Chang X.Y., Dong T.F. (2023). Effect of Alkylation Chain Length on Inhibiting Performance of Soluble Ionic Liquids in Water-Based Drilling Fluids. ACS Omega.

[18] Liu Y., Guo Y., Tang Y., Sun C., Yuan L., Zhang Y. (2021). Synthesis and interfacial properities of sulfobetaine-PEG copolymer. Chem. Res. Appl..

[19] Kang W.L., Zhang H.W., Lu Y., Yang H.B., Zhu T.Y., Zhang X.F., Chen C., Sarsenbekuly B., Besembaevna O.Z. (2019). Study on the enhanced viscosity mechanism of the cyclodextrin polymer and betaine-type amphiphilic polymer inclusion complex. J. Mol. Liq..

[20] Darabi K.A., Ahmadlouydarab M. (2024). Injection of polyacrylamide and polyethylene glycol solutions to assess enhanced oil recovery and drawbacks of the polymer injection. Geoenergy Sci. Eng..

[21] Zhu J.Z., Liang H.J., Liu Y.L., Yu B.Y., Ma C. (2024). Synthesis and Evaluation of High-Temperature Resistant Polymer Plugging Agent for Water-Based Drilling Fluids. ACS Omega.

[22] Yan Y.C., Xue Z.Y., Wu L.C., Luo Y.M., Bai X.D. (2025). Synthesis and Application of a Temperature Sensitive Poly(Acrylamide-Co-N-Isopropylacrylamide-Co-Sodium P-Styrene Sulfonate) as a New Water-Based Drilling Fluid Plugging Agent. J. Appl. Polym. Sci..

[23] Li P.P., Xu Y., Liu Y., Feng J., Hui B.T., Feng Y.K., Hu M.M., Guo J.T. (2021). Terpolymer with rigid side chain as filtrate reducer for water-based drilling fluids. J. Appl. Polym. Sci..

[24] Mohamadian N., Ghorbani H., Wood D.A., Khoshmardan M.A. (2019). A hybrid nanocomposite of poly(styrene-methyl methacrylate- acrylic acid) /clay as a novel rheology-improvement additive for drilling fluids. J. Polym. Res..

[25] Ma J.Y., An Y.X., Yu P.Z. (2019). Core-shell structure acrylamide copolymer grafted on nano-silica surface as an anti-calcium and anti-temperature fluid loss agent. J. Mater. Sci..

[26] Zhang T.F., Sun J.S., Liu J.P., Lv K.H., Sun Y.W., Xu Z., Huang N., Yan H. (2025). High-temperature and high-salinity resistance hydrophobic association zwitterionic filtrate loss reducer for water-based drilling fluids. Pet. Sci..

[27] Wang G.S., Jiang G.C., Yang J., Yang L.L., Li X.L., He Y.B., Chang X.Y. (2021). Novel *N*, *N*-dimethylacrylamide copolymer containing multiple rigid comonomers as a filtrate reducer in water-based drilling fluids and mechanism study. J. Appl. Polym. Sci..

[28] Sun Y.W., Sun J.S., Lv K.H., Liu J.P., Shi C.J., Zhang T.F., Zheng Y.F., Yan H., Li Y.C. (2026). Enhancing acrylamide-based polymer performance in high temperature drilling fluid: Role of isopentenol polyoxyethylene ether. Pet. Sci..

[29] Balaga D.K., Kulkarni S.D. (2022). A review of synthetic polymers as filtration control additives for water-based drilling fluids for high-temperature applications. J. Pet. Sci. Eng..

[30] Li J., Fan J.H., Sun J.S., Wang Z.Y., Lv K.H., Qu Y.Z., Xu G.Q., Ma F.B., Li W., Ma W.L. (2025). A Hyperbranched Copolymer as High-Temperature and Salt- Resistance Fluid Loss Reducer for Water- Based Drilling Fluids: Preparation, Evaluation, and Mechanism Study. SPE J..

[31] Davoodi S., Al-Shargabi M., Wood D.A., Rukavishnikov V.S., Minaev K.M. (2023). Thermally stable and salt-resistant synthetic polymers as drilling fluid additives for deployment in harsh sub-surface conditions: A review. J. Mol. Liq..

[32] Li J.R., Lin L., Li L., Lu H., Xu C.Y., Zhang X.M., Ren W. (2026). Preparation and performance evaluation of self-degrading core-shell structure temporary plugging agent. Pet. Sci..

[33] Yin T.H., Zhu Y.M., Yang Y.Q., Chen S.G., Chen J.R., Lu Y.Y., Guo H.Y., Wan T., Liu J. (2026). Double-crosslinked self-degradable hydrogel for temporary plugging in low temperature reservoirs. Pet. Sci..

[34] Ji R.J., Yu X.R., Xu Z.X., Wang X.Q., Tan J.Y., Han Y.Q., Yang H., Su G.S. (2026). Degradable PAM hydrogel mediated by hydroxy acids for temporary plugging. Pet. Sci..

[35] Gao S.D., Lin D., Li A., Deng L.D., Dong A.J., Zhang J.H. (2024). Synergistic effects of covalent crosslinking and hydrophobic association on enhancing thermal and salt resistance of polymeric filtrate reducer. J. Mol. Liq..

[36] Quan H., Xiao S., Liang Y. (2025). Synthesis and performance evaluation of DASAN filtrate loss reducer for temperature resistant and salt tolerant drilling fluid. Mod. Chem. Ind..

[37] Liu H., Zeng X., Li W., Wang F., Zhang H. (2023). Preparation and properties of a novel temperature-and salt-resistant polymer fluid loss reducer. Mod. Chem. Ind..

[38] Liu X.L., Yuan Z.Y., Wang A., Wang C.P., Qu J.L., Chen C., Wei B., Kapu N.S., Wen Y.B. (2020). Cellulose nanofibril-polymer hybrids for protecting drilling fluid at high salinity and high temperature. Carbohydr. Polym..

[39] Qu Y.Z., Zhang W.C., Ren H., Yuan Y.H., Zhou X.Y., Yang G., Zhang X.F. (2024). Investigation of the correlation between molecular structures with high-temperature and salt resistance for acrylamides polymer in drilling fluid. J. Dispers. Sci. Technol..

[40] Li H., Huang X.B., Sun J.S., Lv K.H. (2023). Multi-ring side groups copolymer as an effective filtration control additive for water-based drilling fluids under high temperature and salt contamination. Geoenergy Sci. Eng..

[41] Koh J.K., Lai C.W., Johan M.R., Gan S.S., Chua W.W. (2022). Recent advances of modified polyacrylamide in drilling technology. J. Pet. Sci. Eng..

[42] Sun J.S., Wang Z.L., Liu J.P., Lv K.H., Zhang F., Shao Z.H., Dong X.D., Dai Z.W., Zhang X.F. (2022). Notoginsenoside as an environmentally friendly shale inhibitor in water-based drilling fluid. Pet. Sci..

